# Diagnosing Burn Wounds Infection: The Practice Gap & Advances with MolecuLight Bacterial Imaging

**DOI:** 10.3390/diagnostics11020268

**Published:** 2021-02-09

**Authors:** Nawras Farhan, Steven Jeffery

**Affiliations:** 1 Burn Centre, Birmingham Women’s and Children’s NHS Foundation Trust, Birmingham B4 6NH, UK; 2 Wound Healing Practice Development Unit, Birmingham City University, Birmingham B15 3TN, UK; sjeffery@nhs.net

**Keywords:** fluorescence, imaging, burns, wounds, bacteria, MolecuLight *i:X*

## Abstract

Burn injuries constitute a critical economic burden on healthcare infrastructures worldwide. They are often associated with high mortality rates due to severe complications. Infection is the most common complication, highlighting the importance of prompt and precise diagnosis in order to prevent detrimental consequences and to optimize patient outcomes. Here we examine the current standard of care for diagnosing infection in both burn and chronic wounds followed by an investigation into the research surrounding a relatively new technique for bacterial detection, fluorescence imaging. With five years of published research on bacterial fluorescence imaging (MolecuLight *i:X* device), we have summarized and analysed the validity of the procedure and compared it to the current standard of care; clinical assessment and microbiological analysis. We highlight the benefits that could be obtained through the use of this technology as well as the limitations and the feasibility of incorporating this novel procedure into the standard of care.

## 1. Introduction

Burn wounds represent a significant clinical and economic strain around the world. The estimated annual NHS cost is £89.6 million to manage 87,000 yearly burns and the associated comorbidities [1]. Infection is the most common life-threatening complication of burn wounds and is associated with up to 75% of mortality among burn victims [2,3]. Thus, infection prevention represents a major concern during the care of burned patients. The instant and accurate identification of burn wound infection is of paramount importance to prevent the cascade of deleterious sequelae [4]. Early detection and intervention when the infection is suspected remains of the utmost importance. Yet, key challenges in this early identification are frequently encountered. 

The current diagnostic approach involves a bed-side visual assessment to detect the Clinical Signs and Symptoms (CSS) of wound infection which may often be supported by semi-quantitative microbiological analysis using wound swabs and culturing. The shortcomings of the former approach lie in its subjectivity and inability to detect clinically significant bacterial levels in asymptomatic patients [5], while the latter requires days to be reported and can be prone to false-negative results [6]. 

This results in a gap in diagnostic tools for clinicians to accurately assess burn wounds for elevated bioburden in real time. However, novel imaging techniques have been developed in an attempt to tackle this issue. Fluorescence imaging allows for the visualization of autofluorescence emission by microorganisms and by the surrounding skin constituents in real time without contrast agents to detect elevated levels of bacteria [7,8]. This review examines the current standard of care for detecting burn wound infections and how the addition of fluorescence imaging may aid in these efforts. 

### 1.1. Pathogenesis of Burn Wounds Infection 

For a better estimation of burn wound infections, the dynamic changes of these wounds at the cellular level should be explored. A certain amount of endogenous skin flora can be tolerated by the intact skin without causing any harmful effects; however, elevated levels of foreign bacteria in a burn injury can cause infection due to the breach in the skin barriers and the associated immunosuppressive state experienced by a burn victim [9]. Deep dermal and full-thickness burns provide an appropriate niche for bacterial habitation and proliferation due to the protein-abundant environment and the presence of avascular necrotic tissues, namely, eschar [2]. The lack of vascularity aggravates the situation by impeding immune cell migration and systemic antimicrobial drug delivery, whereas the eschar tissues still release toxic substances that weaken the local immune response, ultimately resulting in pathogenic invasion [2]. 

Wound contaminants originated mainly from three principal sources: (a) normal commensal microflora from adjacent skin surfaces (*Staphylococcus epidermidis*, skin diphtheroids, etc.); (b) exogenous sources from the environment; and (c) endogenous sources from the host’s mucous membranes (alimentary, respiratory, and genitourinary mucosae) [2,10]. Although the burn wound typically remains sterile for the first 6 to 12 h post-injury, Gram-positive bacteria quickly colonize within the first week of admission [3]. Gram-negative bacteria, such as *Pseudomonas aeruginosa,* may begin to appear and proliferate after this initial week [3]. The causative agents of nosocomial infections vary among facilities and tend to display more antibiotic resistance than those originating from endogenous sources [2]. 

Burn wounds are subjected to colonization not only by bacteria in a free-floating planktonic state but, to a large extent, a biofilm as well. A microbial biofilm is composed of bacterial aggregates that are embedded in a self-produced Extracellular Polysaccharide (EPS) matrix [9,11,12]. This EPS matrix surrounding the bacteria serves many purposes, including strong adherence to the wound bed and protection from environmental factors like desiccation, immune targeting, and antibiotic treatments [11,12]. The bacteria in these biofilms can be up to 1000 times more resistant to antimicrobials than planktonic (free-floating) bacteria [13]. Together, these factors make biofilms increasingly hard to treat. Current wound management practice advocates the concept of biofilm-based wound care (BBWC) established on understanding the biofilm cycle, to break the early stages of attachment and phenotypic changes, and also to halt the reformation process [14].

**Figure 1:**
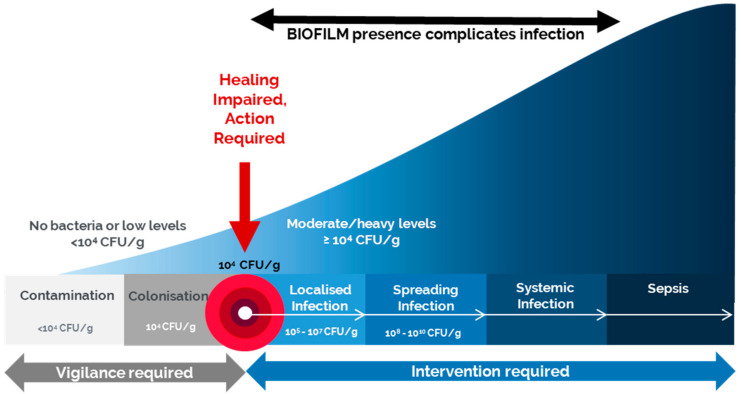
The Wound Infection Continuum. When wound bacterial loads exceed 10^4^ CFU/g, intervention is required to address biofilm and prevent serious infection from occurring. (Modified from Woodmandsey and Roberts. Int Wound J 2018).

The dynamic interaction between the host immune system, the pathogens, and the surrounding environment results in a wound infection continuum with different stages reflecting the diversity of a pathogenic impact on wounds (Figure 1). According to the International Wound Infection Institute (IWII) [14], the stages of this wound infection continuum can be defined as follows:

- **Contamination** refers to the existence of non-proliferating microorganisms at a level that cannot trigger the immune response. Any open wound will contain some contamination with bacteria, typically natural flora, yet these bacteria are non-proliferating and at levels that do not evoke a host response or delay healing [14].
- **Colonization** is the presence of microorganisms with a limited proliferation rate without triggering the immune response or delaying healing. During these stages, vigilance is required but not necessarily antimicrobials [14].
- **Local infection** begins to occur as the bacteria move deeper into the wound, proliferate at a faster rate, and initiate the beginnings of a host response [14]. This infection may present with subtle signs and is essential for early detection and intervention to help prevent further escalation [14].
- **Spreading infection** occurs as the bacteria increase in number and virulence and begin to invade the surrounding tissue and more overt signs of infection present like delayed wound healing, potentially erythema, wound breakdown, and dehiscence [14].
- **Systemic infection** is the most advanced stage which affects the whole body via vascular or lymphatic routes, leading to serious consequences such as sepsis and organ dysfunction [14].

The point at which bacterial loads tip from colonization to local infection is a matter of some debate. While the literature is varied, most studies suggest loads of between 10^4^ CFU/g and 10^5^ CFU/g [10,15,16,17]. For example, a study by Breidenbach and Trager demonstrated that a critical bacterial load threshold of 10^4^ CFU/g must be attained to cause infection in complicated lower-limb wounds [18]. 

The prompt detection of wound infection and high bacterial loads greatly influences wound management and outcomes. It allows prudent use of antimicrobials at early stages to stop the negative sequelae of late diagnosis such as delayed wound healing and helps to reduce the mortality rate [2]. Early intervention is essential to combat bioburden, yet in order to intervene, timely and accurate detection of bacterial burden is required. The current standard of care relies on the assessment of CSS of infection along with some microbiological assessment. Fluorescence imaging has also begun to emerge as an excellent diagnostic tool to support the current standard of care. 

### 1.2. Standard of Care: Clinical Signs and Symptoms

The current standard of care for many wounds is a visual wound assessment, which includes an assessment of CSS of infection. The IWII has developed a checklist that involves the detection of covert “subtle” signs as well as overt “classical” signs of infection (Table 1) [14]. These markers of infection may include such signs and symptoms as new, increased, or altered pain; delayed healing; peri-wound oedema; bleeding or friable granulation tissue; odour; wound bed discolouration; purulent exudate; induration; pocketing; bridging. However, detecting those signs may be challenging. Assessment of these symptoms is subjective and variable across care providers, often based on their level of specialized training or extensive experience. Furthermore, these symptoms may vary or be less obvious in patients who are immunocompromised or have motor or sensory neuropathies [19]. Burn wounds, in particular, present a challenge because multiple systemic indicators of infection (fever, hypotension, elevated peripheral blood white blood cell count, etc.) can be quite common in uninfected burn patients [3]. While a newly developed or increasing onset of pain is considered as an indicator of infection, pain proprioception is impaired in deep-dermal and full-thickness burns. Alternatively, erythema could be presented as a consequence of a burn injury as opposed to infection [20]. 

Numerous studies have reported that patients with a high bacterial burden are frequently asymptomatic [5,21,22,23]. A meta-analysis of 15 clinical studies evaluating the effectiveness of various CSS in 1056 chronic wounds found pain to be the only useful sign or symptom in diagnosing infection [5]. Gardner et al. revealed no significant correlation between CSS and wound infection [21], and Le et al. have also demonstrated the poor discriminatory power of CSS in detecting bacteria [7]. Serena et al. highlighted issues surrounding subjectivity, an inability to identify “subclinical infection,” which occurs when bacterial levels reach a critical load without manifesting any CSS, and the need for long-term evaluation to confirm the presence of some signs like delayed wound healing [22]. All of these factors hinder the immediate identification of wounds with a high bacterial burden and may result in delayed interventions. 

### 1.3. Standard of Care: Microbiological Assessment

Clinical assessment is often augmented by microbiological investigations to determine prominent bacterial species and possible resistance genes present in these wounds. These microbiological samples are customarily taken only when the infection is already suspected and typically act as a confirmation of CSS assessment rather than an independent diagnostic tool; however, some experts have advocated for performing routine infection surveillance of burn wounds using swab cultures [2,3]. The most commonly used methods include either swab sampling (Levine or Z technique) or tissue sampling (biopsy or curettage), with needle aspiration sampling being quite rare [6,24,25]. 

Obtaining swabs is often the preferred method of sample collection due to its cost-effectiveness and the fact that it is non-invasive and less time-consuming [26]. The Levine technique is the preferred swabbing technique, in which a 1 cm^2^ area of the wound is swabbed and the wound is probed to express some wound exudate [6,27]. However, evidence shows that swab sampling is superficial and may not reflect the bacterial presence on deeper tissue levels leading to results that represent only surface contamination and colonization [10]. Literature suggests the benefits of deep tissue sampling compared to surface swabbing [26,28]. Reports have shown that tissue samples such as curettage and punch biopsies detect more bacterial pathogens, both in the number of species and total bacterial loads, which may better represent the causative pathogens [26,28].

However, regardless of the sampling method, microbiology results are not obtained in real time, often requiring 2 to 5 days before the results are reported [6]. This may cause a delay in early wound care interventions to combat the bioburden which may be altered by the time the results are reported. In addition to the variability in sampling methods, the type of microbiological assessment and how the results are reported can also have a profound impact on their quality. PCR analysis has been shown to be superior to culture analysis in detecting additional bacterial species [29,30], yet increased costs and specialized equipment mean that most institutions continue to rely on culture-based microbiological analysis. One of the drawbacks of culture analysis is that many anaerobic or fastidious bacterial species can be overlooked and underreported [10].

Furthermore, culture results may be reported in either a semi-quantitative or a quantitative manner. While guidelines recommend that wounds be treated based on quantitative cultures reported in CFU/g [14], semi-quantitative cultures, which report bacterial loads in no, occasional/scant, light, moderate and heavy growth per culture dish, remain commonplace. The relationship between these quantitative and semi-quantitative culture reports remain to be fully elucidated. One study suggests that each of these semi-quantitative levels could represent up to a 4 log spread of bacterial loads reported in CFU/g [31]. More research is required to better understand the differences in quantitative and semi-quantitative culture results and how that may affect treatment plans. 

Understanding the bacterial burden of wounds, however, considers an important aspect in determining care. Multiple studies have indicated that bacterial loads of greater than 10^4^ CFU/g contribute to delayed wound healing [15,17]. Additionally, failure to reduce the bacterial burden prior to grafting has been shown to reduce graft take to <20%, often resulting in a complete loss of the graft [32]. Another study highlighted the presence of *Pseudomonas aeruginosa* as a major risk factor for graft failure [33]. 

These and many other studies stress the importance of understanding the presence of a significant bioburden in a wound to guide treatment decisions. The clinical issue that persists is what tools clinicians can use to determine this bioburden at the patient bedside. As discussed, while necessary to the current standard of care, CSS assessment can be very subjective and microbiology assessment takes days for the result. A new technology, fluorescence imaging, fills this gap by providing real-time information on the bacterial presence in a wound. 

## 2. Fluorescence Imaging with MolecuLight *i:X*

The use of fluorescence-based clinical tools has been well established, and the clinical utility of one particular fluorescence imaging device, the MolecuLight *i:X,* has been described in numerous trials and publications [7,34,35,36,37]. Fluorescence imaging utilizes the basic principles of tissue and bacterial autofluorescence, which is the property of fluorescent molecules (fluorophores) absorbing a wavelength of light and then emitting a longer wavelength of light [38]. Specifically, when using violet light excitation to image wounds or other skin conditions, the two main substances that fluoresce are dermal connective tissues and porphyrins [38,39,40,41,42]. Bacteria imaged under violet light excitation may produce fluorescence through either endogenous porphyrins [39,43,44,45] or pyoverdines [46,47]. These fluorescence signatures can be observed and documented to provide real-time information on the bioburden. The principal fluorescence imaging device that has been clinically validated is the MolecuLight *i:X.*

**Figure 2:**
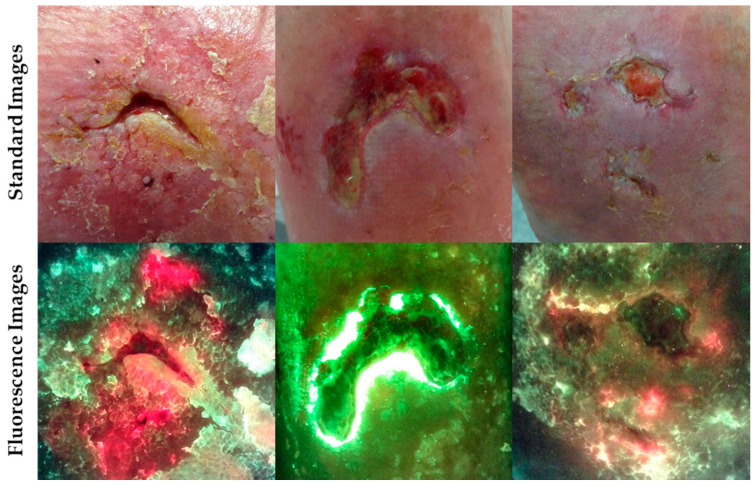
Examples of MolecuLight standard and fluorescence images. The MolecuLight *i:X* takes both standard and fluorescence images. These are 3 examples of wounds imaged with the MolecuLight. In fluorescence images, red or cyan fluorescence indicate the presence of bacterial loads >10^4^ CFU/g or moderate-to-heavy loads. (Left) Venous leg ulcer with red fluorescence. Microbiology: 2 × 10^4^ CFU/g. (Center) Venous leg ulcer with cyan fluorescence. Microbiology: 6 × 10^6^ CFU/g. (Right) Venous leg ulcer with red fluorescence. Microbiology: 5 × 10^7^ CFU/g.

In order to perform fluorescence imaging, in a darkened room, the MolecuLight ***i****:X* shines a safe violet excitation light (405 nm) on a wound causing wound components (skin, slough, blood, bacteria, etc.) to fluoresce different colours [8,34,35]. The MolecuLight ***i****:X* displays and captures images of only the most informative of these fluorescent colours. Green fluorescence from the skin provides the anatomical context. Red and cyan fluorescence are associated with regions of bacteria. Clinical trials in which curettage or biopsies were collected from wound regions positive for red or cyan fluorescence for gold standard quantitative culture analysis consistently revealed bacterial loads of ≥10^4^ CFU/g [8,34], which is typically moderate-to-heavy growth [8,34,35] corresponding with the presence of red or cyan fluorescence signals. At these loads, most bacterial species are indicated by red fluorescence based on their ability to produce porphyrins, while cyan fluorescence is indicative of *Pseudomonas aeruginosa* [8,35,48]. Figure 2 shows a few examples of these fluorescence images, with areas of bacterial fluorescence noted. 

Due to the endogenous autofluorescence, no exogenous contrast agents are needed during imaging. There is no contact with the patient whatsoever; therefore, the compromised burn patient is not at any additional risk from imaging. In a study visualizing paediatric burn wounds when clinicians were asked about the ease of the procedure, 93% of clinicians indicated very high practicality of use in the routine clinical practice, with the remaining 7% indicating high practicality [49]. Another study reported that fluorescence imaging contributed to 90% improvement in patient care [7]. 

Several trials investigating the diagnostic accuracy of fluorescence imaging using the MolecuLight *i:X* device have placed the positive predictive value of red or cyan fluorescence in detecting bacterial loads of ≥10^4^ CFU/g at over 95% [7,34,35,36,37]. The following in-depth analysis of the diagnostic accuracy measures of this device truly supports the use of fluorescence imaging in detecting clinically significant bacterial loads. The following case study represents an interesting case of an infectious cancerous growth on which fluorescence imaging was performed. 

### Case Study 1

**Figure 3:**
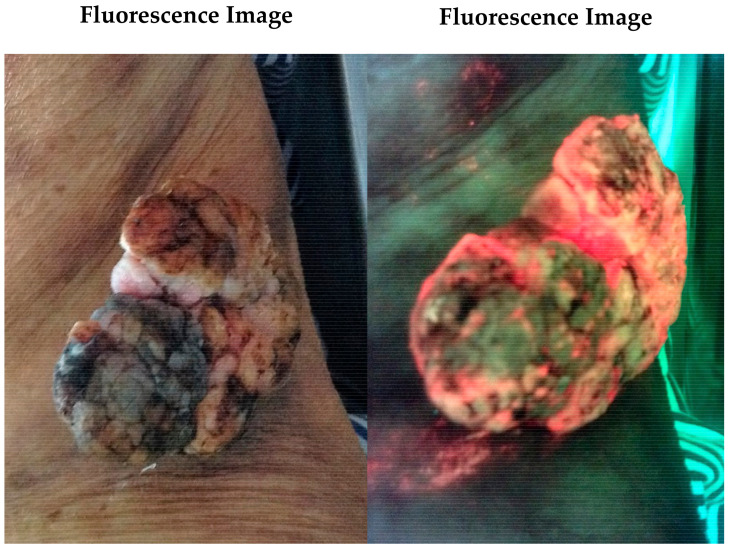
Case Study 1—Infected Cancerous Lesion. Fluorescence imaging of this skin cancer growth demonstrated areas of red bacterial fluorescence. Before excision of the growth, image-informed cleaning was performed to maximally remove bacterial loads and avoid wound infection. Microbiology: *Staphylococcus aureus.*

In this case study, a seventy-two-year-old man came to the clinic with a six-month history of a skin tumor on his thigh (Figure 3). The lesion required excision to remove skin cancer. Fluorescence imaging with the MolecuLight device showed a large amount of red bacterial fluorescence present on the surface of the growth. The lesion was swabbed and the results arrived a few days later to report the growth of *Staphylococcus aureus.* However, based on the real-time results of the fluorescence imaging, disinfection of the lesion was carried out preoperatively to prevent subsequent wound infection. The lesion was then excised and the excision wound healed uneventfully. 

The importance of good wound hygiene in wound care is very well known. The ability to visualize bacterial fluorescence at the point of care allowed for the timely and effective cleaning of this lesion. This was particularly important prior to excision, as residual bacterial loads could have colonized the wounds and eventually caused serious complications. By performing good wound hygiene before excision and monitoring the bacterial burden throughout the healing process, the excised wound healed completely without complications. 

## 3. Diagnostic Accuracy of Fluorescence Imaging

As fluorescence imaging for bacterial detection has gained acceptance in the field, various groups have studied the diagnostic accuracy of the MolecuLight *i:X* in various wound types and care settings. A landmark paper by Rennie et al. described a bacterial load threshold of ≥10^4^ CFU/g in areas of red fluorescence when wounds were imaged with the MolecuLight *i:X* with a 100% positive predictive value (PPV) [34]. This qualification of the device’s threshold of detection was supported by data from 60 patients with samples taken by curettage scraping or punch biopsy [34]. 

To date, 11 publications, representing a total of 613 wounds, have reported on the diagnostic accuracy measures of the MolecuLight *i:X* to detect bacterial loads of ≥10^4^ CFU/g (Table 2) [7,35,36,37,49,50,51,52,53,54,55]. The diagnostics accuracy measures reported here include the sensitivity, specificity, positive predictive value (PPV), negative predictive value (NPV) and accuracy. Sensitivity measures the probability of the test to correctly identify a wound with elevated bacterial loads above 10^4^ CFU/g, while specificity examines the portion of patients without elevated bacterial loads that have a negative result. PPV represents the proportion of wounds with red or cyan fluorescence that have elevated bacterial loads ≥10^4^ CFU/g, while NPV represents the proportion of wounds without elevated bacterial loads that would get a negative result. These are based on false positives and false negatives, respectively. Accuracy is defined as how well the test correctly identifies a wound with or without elevated bacterial levels, taking into account both false positives and false negatives. 

These publications span the field in terms of wound type and care settings. Only 4 of these papers specifically focus on burn wounds; however, it is encouraging to understand that the diagnostic accuracy measures of this technology are applicable across many wound types. Table 2 reports a meta-analysis of these 11 papers and indicates a significant sensitivity of the device at detecting these bacterial loads (average 89%, weighted average 74%) with a weighted average positive predictive value of 91% and an accuracy of 75%. 

While these diagnostic accuracy values do represent a significant improvement over the current standard of care, there is a clear variability between studies, which bears further analysis. In addition, while this analysis may bring additional insight into the use of fluorescence imaging, it is important to realize that the majority of these studies are observational with small sample sizes. Only Le et al. represents a large statistically powered study [7]. This should be born in mind during the following discussion as the small sample size of some of these studies may artificially inflate or diminish the diagnostic accuracy measures reported in the studies. Of particular note, the small sample size and single true negative sample reported in Serena et al. suggests that the NPV and specificity values be interpreted with caution [36]. Furthermore, Blumenthal et al. classified any microbiology result (event scant growth) as microbiologically positive despite the stated detection threshold of the MolecuLight *i:X*, which artificially increased false-negative results and lowered the NPV [55]. Image interpretation experience may also be a factor in these measures, as it has been reported that the fluorescence image requires a clinician’s interpretation of the image, which has an established learning curve [8]. 

Interestingly, the studies that focused on burns reported better diagnostic accuracy measures compared to the average of all the papers. A sub-analysis of the 4 burn studies [49,53,54,55] demonstrated higher sensitivity (91%), accuracy (85%) and NPV (82%) with only minor decreases in PPV and specificity. This might suggest that the burn population, in particular, would see a large benefit from fluorescence imaging. 

While these diagnostic accuracy measures provide important information about the general utility of fluorescence imaging as a procedure, two major elements should be considered to place these values in context. The first is how these measures compare to the current standard of care, which is the assessment of CSS of infection. The second major factor to consider is the sampling method, whether the microbiology sample was taken via swab or biopsy, which contributes to some of the differences in these diagnostic measures.

### 3.1. Comparison of Fluorescence Imaging to Clinical Signs and Symptoms

As previously mentioned, the assessment of CSS of infection is the current standard of care for determining wounds likely harbouring clinically significant levels of bacteria at the point of care. However, these clinical signs and symptoms can be extremely subjective or simply absent, especially in patients with other co-morbidities, leading to very low sensitivity in detecting wounds with clinically significant levels of bacteria [5,19,21,22,23]. Indeed, one recent paper examined the sensitivity and specificity of each of the CSS listed in the IWII guidelines [14] and found that all but one were extremely poor predictors of bacterial load [7]. The one exception was delayed healing, which had reasonably high sensitivity but poor specificity, indicating that while high bacterial loads often delay wound healing, there are other causes for delayed healing as well [7]. Burns are complex wounds that may further complicate the detection of these CSS, with impaired pain proprioception and burn-related erythema.

Placing the diagnostic accuracy measure of fluorescence imaging in the context of the current standard of care is critical to appreciate the benefits of such a procedure. Only one publication has directly assessed the accuracy of CSS compared to fluorescence imaging in the context of burns. Blackshaw et al. reported a sensitivity of 63% for CSS, while fluorescence imaging increased the sensitivity to 100% [54]. This results in a 59% increase in sensitivity when fluorescence imaging was used compared to CSS assessment alone [54]. This is a sizable increase in sensitivity, resulting in the detection of more wounds harbouring bacterial burdens that were left undetected by CSS assessment. While this is a notable result, consideration must be given to the relatively modest sample size of only 14 wounds in this study [54]. 

However, this increase was dwarfed by the increase in sensitivity observed in other studies examining chronic wounds of mixed etiology. Le et al. and Serena et al. were successful in demonstrating a huge improvement in the sensitivity and accuracy of bacterial detection based on fluorescence imaging [7,36]. Both studies utilized punch biopsy tissue samples to determine the microbiological loads of the wounds and a CSS checklist to determine bacterial presence based on the standard of care [7,36]. Their results pointed to a 3- to 4-fold increase in the sensitivity when fluorescence imaging was used compared to CSS assessment alone [7,36]. In both studies, the sensitivity of CSS assessment was very low (15% and 22%), and fluorescence imaging detected between 45% and 47% more wounds with clinically significant levels of bacteria that the CSS assessment missed [7,36]. 

Other papers demonstrated a more subtle yet still remarkable and statistically significant effect. Hill et al. reported a more robust sensitivity and accuracy for their CSS assessment tool, UPPER/LOWER checklist with 82% and 85%, respectively [37]. Yet, fluorescence imaging still increased this 17–22% to 100% accuracy and sensitivity [37]. Jones et al. reported a 60% sensitivity and accuracy of CSS assessment in their analysis, compared to 100% sensitivity and accuracy of fluorescence imaging [52]. Again, this was a 66% increase in both sensitivity and accuracy when fluorescence imaging was used compared to CSS assessment alone [52]. In all of these studies, additional wounds were detected that had been missed by CSS assessment alone [37,52,54]. Other studies have also suggested that CSS assessment has poor discriminative power to predict wounds with high bacterial loads compared to fluorescence imaging. Hurley et al. reported that only 21% of the study patients had overt CSS while 95% of the swab samples were positive for significant bacterial growth [35]. 

Differences in the patient population and sampling methods may account in part for the higher specificity of CSS in these studies. Serena et al. and Le et al. both examined outpatient wound care centre patients using punch biopsy tissue samples [7,36], while Hill et al., Jones et al., and Blackshaw et al. used swab-based sampling and focused primarily on hospital inpatient, long-term care, or burn and trauma patients, respectively, where patients are generally more compromised and potentially more likely to mount symptoms of infection [37,52,54]. The effect of the sampling method appears to play a significant role in the diagnostic accuracy, whether to a benefit or detriment, despite the vast increase as compared to CSS.

### 3.2. The Role of Sampling Techniques in Fluorescence Imaging Diagnostic Accuracy

The method by which a sample is taken for microbiology can have profound effects on the microbiological data received. The most commonly used methods include either swab sampling (Levine or Z technique) or tissue sampling (biopsy or curettage) [6,24,25]. As described above, there is much debate over the use of swab versus tissue sampling for microbiological investigations. The ease of swab sampling is often preferred, despite reports that indicate tissue samples detect more bacterial pathogens, both in the number of species and total bacterial loads, which may better represent the causative pathogens [26,28].

Concerning the diagnostic accuracy measures, studies that relied on swab sampling demonstrated a much higher sensitivity, accuracy, and NPV for MolecuLight *i:X* to detect these moderate to heavy (>10^4^ CFU/g) bacterial loads. The weighted averages of those 9 papers (Table 2) increase the sensitivity of MolecuLight *i:X* to 95%, accuracy to 91%, and NPV to 80%. However, this was associated with a decrease in specificity to only 74% and PPV to 84%. This decrease is not unexpected as the violet light can penetrate up to 1.5 mm through the skin, potentially alerting clinicians to subsurface bacterial loads that would not be detected using a swab sampling method. Conversely, sampling via tissue biopsy results in a very high PPV of 96% [7], indicating a very low false positive for fluorescence imaging. This corresponds to the collection of subsurface bacteria from this sampling method, ensuring that the subsurface bacteria detected by fluorescence imaging is also detecting in the microbiology report. However, this increase in PPV is associated with a decrease in sensitivity, NPV, and overall accuracy [7]. Again, this finding is not necessarily unsurprising. A punch biopsy will obtain a much deeper sample than a swab, including bacterial presence that may exceed 1.5 mm in depth. However, independent of how these diagnostic accuracy measures change based on the sampling method, it is important to recall the overall increase in diagnostic accuracy compared to the current standard of care. 

Another important point to consider when evaluating sampling methods is the location at which the sample is taken. One limitation of the Levine technique is that it avoids sampling the wound edge [27], yet multiple studies, including those using MolecuLight *i:X* for fluorescence imaging, have noted increased bacteria growth specifically in the peri-wound [49,57,58]. Furthermore, tissue samples are taken from specific regions in a wound; biopsies taken from different parts of the same wound will garner different microbiology results in a higher percentage of cases [59]. The use of fluorescence imaging to guide sampling location, whether via swab or tissue sample, has been well reported [49,50,58]. One pilot evaluation compared standard Levine swab results with fluorescence-guided curettage samples and found that the Levine technique gave a 36% false-negative laboratory report [58]. Another paper on paediatric burns also demonstrated incidences of false-negative laboratory results, burns which were positive under fluorescence imaging and by CSS assessment but returned negative microbiological results due to the incorrect sampling location in 50% of an albeit small sample size [49]. The costs of false-negative microbiology reports extend further than the wasted cost of the sample and include the cost of delayed treatment of these potentially infected wounds. Based on the high PPV reported in these multiple studies on the diagnostic accuracy of fluorescence imaging (Table 2), fluorescence-guided wound sampling can increase the likelihood of true positive microbiology results and help to reduce or eliminate false-negative microbiology reports by sampling in locations of red or cyan fluorescence. 

## 4. Fluorescence Imaging Shines a Light on Wound Microbiology

Not only has the compendium of literature on fluorescence imaging aided in validating the technology, but much of the research has also expanded our understanding of the wound microbiome. Many of these papers report the diagnostic accuracy of fluorescence imaging, and also the microbiology culture or PCR results. A recent publication investigated 32 of the most common bacterial species found in chronic wounds to determine if they were capable of producing porphyrin-specific red fluorescence under in vitro experimental conditions [56]. In this study, 28 of the 32 bacterial species tested produced detected porphyrin-specific red fluorescence in the in vitro assay [56]. Of those 28 red-fluorescing species, 22 have been detected in clinical studies in areas of red fluorescence, as shown in Table 3. 

These data highlight the prevalence of the mentioned bacterial species in wounds, as many of them were reported in several publications on fluorescence imaging; *Staphylococcus aureus, Pseudomonas aeruginosa,* and *Klebsiella pneumoniae* being the most often identified. When examining the burn-related publications, *Staphylococcus aureus* and *Pseudomonas aeruginosa* were still the most common bacterial species, having been detected in all 5 burn papers examined [49,54,55,62,63]. This is in agreement with reports that mentioned *Staphylococcus aureus, Pseudomonas aeruginosa, Acinetobacter baumanii,* and *Stenotrophomonas maltophilia* as the most concerning pathogens in burn wounds due to their tendency towards antibiotic resistance [3]. *Pseudomonas aeruginosa* is a particularly interesting bacteria as it produced red fluorescence in the in vitro assay described in Jones et al. [56], yet clinically typically appears as cyan fluorescence in wounds [8,35,37]. This may largely be attributed to the production of fluorescent virulence factors such as pyoverdine by *Pseudomonas.* Clinically, the abundance of this cyan fluorescent virulence factor may potentially overpower the red porphyrin fluorescence observed in vitro, contributing to the appearance of cyan fluorescence [46,65].

These data also support the extension of the in vitro assay result [56] to the clinical setting, as these bacterial species are also being detected clinically in areas of red or cyan fluorescence signatures. Considering the very large sample size (350 wounds, 1053 bacterial isolates) and the collection of tissue samples for microbiology, it is perhaps predictable that Le et al. reported the presence of almost all the common bacterial species listed in Table 2, within the sampled wounds [7].

Conspicuously absent from the list of prevalent wound bacteria are the common bacterial species *Streptococcus agalactiae, Enterococcus faecalis,* and *Finegoldia magna* as they were among those tested in vitro but did not produce red fluorescence [56]. It is well known that the *Streptococcus* and *Enterococcus* bacterial genera lack the ability to synthesize heme (and thus porphyrins) and instead rely solely on heme uptake [66,67]. Thus, the lack of red fluorescence from these species is expected. While this is a clear limitation of the technique, most chronic wounds are known to be polymicrobial and in the largest study of wound microbiota to date (2963 wounds, analysed via 16S rDNA pyrosequencing), these non-porphyrin-producing bacterial species appeared mono-microbially less than 1% of the time [68]. In fact, where the data were available, a number of the studies listed above highlighted the presence of *Enterococcus faecalis* and several *Streptococcus* species in regions of red fluorescence in combination with additional porphyrin producing bacterial species [36,55,60,61].

This in vitro analysis of porphyrin-producing bacteria and their ability to produce red fluorescence also examined that ability in various yeast species common in chronic wounds. The majority of the yeast species tested did not produce red fluorescence in the same assay or that fluorescence was at a much lower level [56]. Further confirming this, while a couple of the papers listed above did report the yeast species *Candida albicans* in the microbiology reports of red fluorescence positive wounds in combination with bacterial species [34,55], none identified the yeast species as the sole microorganism present in an area of red fluorescence. This supports the idea that the red fluorescence is truly coming from the elevated bacterial loads. 

These data demonstrate the utility of fluorescence imaging in detecting a significant number of bacterial species, based both on in vitro experimental research and through clinical trials. This knowledge can be used to inform treatment plans and sampling location, to provide improved wound care for the patients. 

## 5. Impact of Fluorescence Imaging on Wound Care, Including Burns

The evidence supports the ability of this fluorescence imaging device (MolecuLight *i:X)* to identify a minimum bacterial load of 10^4^ CFU/g [7,34] heralding the possibility of early detection of clinically significant bacterial burdens. However, detection alone is not the major goal in wound care, but the action taken based on this information. Studies have shown that fluorescence imaging can prompt changes in proposed treatment plans including alterations in antimicrobial prescribing [61,69], decisions around negative pressure wound therapy [60], and timing of grafting or applications of skin substitutes [62,70]. Studies have specifically shown that fluorescence imaging can change treatment plans, in one study in as many as 73% of cases [7,36]. Even more encouraging is that the use of fluorescence imaging to inform the location and extent of debridement to target bacterial habitation in recalcitrant wounds has been shown to increase healing rates [71], which is the ultimate goal for most wound care. Other groups illustrated that the overall incorporation of fluorescence imaging into patient care can change the trajectory of wound healing, leading to interventions that placed non-healing wounds on a healing trajectory [72] or healed more wounds altogether [73]. This includes a blinded assessment randomized controlled trial, the goal standard for determining the effectiveness of an intervention. 

Looking specifically at work done in the burn population, Blumenthal and Jeffery compared the fluorescence images with the results of the conventional swabs to assess the feasibility of using the MolecuLight i:X device in diagnosing adult burns infection [55]. Two interesting cases demonstrated the bacterial presence in clinically irrelevant wounds. In the first case, *Staphylococcus aureus* bacteria were detected in the healing tissue opposite to the sloughy aspect while in the second one, the bacteria inhabited deeply within the wound’s folds, unreachable by the swab technique [55]. These are two examples of wounds with significant levels of bacteria, yet did not display classic CSS of infection. With the new fluorescence information, the treatment plans of these patients were changed. Another interesting case was published in the first study which assessed the use of the MolecuLight *i:X* in paediatric burn wounds. In that case, fluorescence imaging detected various gradients of red fluorescence, indicating elevated levels of wound bacteria, in a clinically symptomatic patient with positive leukocytosis, despite a negative wound culture. Fluorescence imaging also has been used in evaluating the efficiency of wound management protocols by comparing pre- and post-intervention images, promoting patient engagement and awareness, also providing insight as to the timing and location of grafting procedures to limit the possibility of graft failure due to inadequate wound preparation prior to surgeries [62].

The use of fluorescence imaging to guide the timing of skin grafts and skin substitutes is of increasing interest as these procedures can be incredibly costly and graft failure all too common. Some research has suggested that the use of fluorescence imaging can influence the timing of these grafting procedures, to a patient’s benefit [62,70]. Below is such an example, where fluorescence imaging revealed the need for additional debridement of a burn wound prior to a skin graft application, with images captured again after debridement to determine whether the bacterial burden was removed. 

Outside of fluorescence imaging, it should be noted that the MolecuLight *i:X* device also has a feature that enables digital measurement of the area, length, width of wounds, and documentation on the image of these measurements as well as a manually measured depth of wounds. The measurement software quickly and automatically measures the surface area of the wound and provides the length and width, with >95% accuracy [58]. 

### Case Study 2

**Figure 4:**
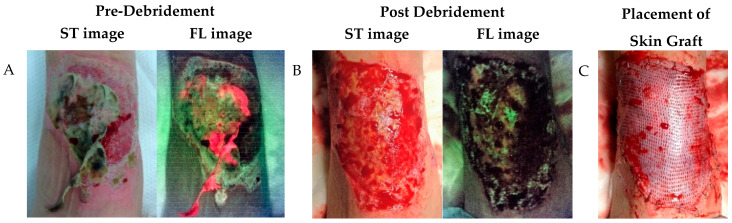
Case Study 2—Debridement of a Burn Wound. (**A**) Pre-debridement, red bacterial fluorescence is observed through the burn and within the slough. (**B**) Post-debridement the de-vitalized tissue and bacterial burden is largely removed. Red bacterial fluorescence is no longer observed. (**C**) Post-debridement and after confirmation of reduced bacterial load, a skin graft was applied to the burn.

A 67-year-old man sustained a flame burn to the leg. Initial MolecuLight *i:X* images demonstrated areas of red bacterial fluorescence throughout the wound and slough (Figure 4A). Based on the MolecuLight fluorescence information, the burn was excised using a Watson knife in the areas of red bacterial fluorescence. After this debridement, another image was taken to confirm the removal of bacterial bioburden (Figure 4B). Immediately after these images were taken, a split-thickness skin grafting was performed (Figure 4C). At the one-week follow-up appointment, a full graft take was seen. 

As skin grafts are contraindicated in the presence of elevated bacterial loads, the ability to monitor the levels of bacterial bioburden in real time allows for additional confidence in the appropriate timing of the spilt-thickness skin graft. Placing a skin graft on a wound with elevated bacterial loads can cause graft failure, which can be costly both to the patient in terms of delayed healing and potential additional complications as well as the health system in terms of materials and the care provider time. With the real-time information on the bacterial burden from the MolecuLight, the clinician had more confidence that they had appropriately prepared the wound bed to receive the skin graft. Indeed, the skin graft took and the wound healed properly.

## 6. Limitations of Fluorescence Imaging: Another Tool in the Toolbox

Despite the benefit fluorescence imaging can provide in burn and wound assessment, there are several limitations that users should be aware of. Fluorescence imaging requires adequate darkness to capture optimal fluorescence images. Ambient light contamination can result in inappropriate image interpretation, which can influence patient care [8]. This requisite darkness can pose a major challenge in certain settings due to large windows or automated lighting system, as has been noted in several publications [35,49,54]. However, solutions can be found including the use of a DarkDrape attachment, which is available to provide the required darkness. 

Another obstacle that can be overcome is a learning curve related to fluorescence image interpretation. In particular, new users may find it difficult to discriminate between the cyan fluorescence from *Pseudomonas aeruginosa* and the green fluorescence from endogenous structures. As with most imaging modalities, continued use of the device and utilization of image interpretation resources [8] can help tremendously in overcoming this learning curve. Good imaging practices are also necessary, such as cleaning the wound, removing as much blood as possible, and removing imaging artifacts from white bedsheets and gauze bandages when possible [8]. Blood in particular can absorb the violet excitation light and mask other fluorescence signatures if not removed [8]. The inability of colour-blind people to accurately interpret the fluorescence images due to the high proportion of red and green colours should also be explored. This represents an area of improvement for the technology, perhaps to develop a “colour-blind mode” for this population. 

Aside from the limitations surrounding the clinical use, fluorescence imaging technology has certain ingrained limitations. The first is that the violet excitation light is unable to penetrate more than 1 mm to 1.5 mm into the skin [74,75]. While this enables the detection of some subsurface bacteria, the presence of bacteria deeper within the wound tissue may not be visible, including deep tunneling infection. Furthermore, the fluorescence signals associated with bacteria cannot provide a numerical estimate for the bacterial load other than indicating it is ≥10^4^ CFU/g. Nor is it able to determine the specific bacterial species or the antibiotic susceptibility of these microorganisms. This would require microbiological analysis using swab or tissue samples. Finally, there are certain bacterial and yeast species that are not detectable when colonizing a wound on their own as described above. 

For these reasons, fluorescence imaging should not take the place of the current standard of care which involves the use of CSS supplemented with microbiological analysis when necessary, but instead acts as an additional tool in the clinician’s toolbox for diagnosis. Using fluorescence imaging can vastly improve wound assessment when used together with CSS, as has been repeatedly shown [7,36,37]. When the microbiological analysis is required to identify specific bacterial species, quantitative loads, and/or resistance markers present, then fluorescence imaging can aid microbiological sample collection analysis by guiding sampling location to a region positive for red or cyan fluorescence to avoid false-negative culture reports [49,50,58]. 

## 7. Conclusions

In conclusion, this review of all current literature on fluorescence imaging has highlighted the multiple benefits of this technique as well as some of the limitations. The field of literature had certainly been expanding in the past few years with this regard, as many publications coming out providing new and corroborating evidence in support of this novel approach Though there are some limitations, fluorescence imaging is increasingly coming to the forefront of the field as a diagnostic tool to be used alongside the existing standard of care. Further research, specifically on healing rates surrounding the use of fluorescence imaging, should be watched for with great interest. 

## Figures and Tables

**Table 1:** International Wound Infection Institute (IWII) checklist of clinical signs and symptoms of infection.

Local Infection				Spreading Infection	
Covert (Subtle Signs)		Overt (Classic) Signs			
Hypergranulation (excessive “vascular” tissue) Epithelial bridging and pocketing in granulation tissue Wound breakdown and enlargement Delayed wound healing beyond expectations New or increased pain Increasing malodor		Erythema Local warmth Swelling Purulent discharge Delayed wound healing beyond expectations New or increasing pain Increased malodor		Extending induration Lymphangitis Crepitus Wound breakdown/dehiscence with or without satellite lesions Malaise/lethargy or non-specific general deterioration Loss of appetite Inflammation, swelling, or lymph glands	
**The number of covert signs present:**	**/7**	**The number of overt signs present:**	**/7**	**The number of spreading signs present:**	**/7**

**Table 2:** A meta-analysis of publications reporting diagnostic accuracy measures of fluorescence imaging for detecting wound bacteria. Microbiology was used to confirm true bacterial loads. n, number of patients in the study; DFU, Diabetic Foot Ulcer; PU, Pressure Ulcer; VLU, Venous Leg Ulcer; SS, Surgical Site; PPV, Positive Predictive Value; NPV, Negative Predictive Value.

Author	n	Study Design	Wound Types	Care Setting	Sampling Method	Sensitivity (%)	Specificity (%)	PPV (%)	NPV (%)	Accuracy (%)
Le et al. [7]	350	multi-center controlled, observational clinical trial	DFU, PU, VLU, SS, other	outpatient wound care centres	biopsy	59	89	96	32	64
Chew et al. [51]	35	observational study	hand trauma wounds	outpatient	swab	100	97	67	100	97
Jones et al. [56]	36	multi-site observational study	DFU, PU, VLU	long-term care	swab	100		94		94
Hill et al. [37]	43	multi-center prospective observational	DFU, PU, VLU, SS, other	inpatient, outpatient	swabs	100	100	100	100	100
Hurley et al. [35]	33	single-center prospective observational	lower-limb wounds	outpatient	swabs (43)	100	78	95	100	96
Serena et al. [36]	19	single-center prospective observational clinical trial	VLU, DFU	advanced-wound care centres	biopsy	73	100	100	17	74
Farhan & Jeffrey [49]	16	observational study	burn	pediatric burns outpatient centre	Levine swabs	100	72	63	100	82
Alawi et al. [53]	14	pilot observational study	burn	not reported	swabs	87	88	82	90	87
Blackshaw & Jeffrey [54]	14	observational study	burn, trauma	burns outpatient department	swabs	100	89	89	100	94
Blumenthal & Jeffrey [55]	20	observational study	burn	burns outpatient department	swabs	81	75	93	50	80
Ottolino-Perry [50]	33	non-randomised clinical trial	DFU	wound care centre	swabs	78	78	64	88	78
**Average**						**89**	**87**	**87**	**78**	**86**
**Weighted Average**						**74**	**88**	**91**	**53**	**75**

**Table 3:** Analysis of bacterial species reported in publications that assessed MolecuLight *i:X* fluorescence imaging. All microbiology samples were taken from wounds exhibiting red or cyan fluorescence. The list of bacterial species represents bacterial species confirmed to produce porphyrin-specific red fluorescence in vitro [56].

Detected in Vitro (Based on Red Porphyrin Fluorescence)		Detected in Clinical Studies (Based on Sampling in Areas of Red or Cyan Fluorescence)
Genus	Species	
***Staphylococcus***	*aureus*	[7,8,34,35,36,37,49,50,54,55,58,60,61,62,63,64]
	*epidermidis*	[7,50,58]
	*capitis*	[7]
	*lugdunensis*	[7,34,35,37]
***Pseudomonas***	*aeruginosa*	[7,8,35,36,37,49,54,55,58,62,63,64]
	*putida*	
***Escherichia***	*coli*	[7,37,50,55,58,61,62]
***Corynebacterium***	*striatum*	[7,36]
***Proteus***	*mirabilis*	[7,8,34,35,52,58,62,63]
	*vulgaris*	[7,60]
***Enterobacter***	*cloacae*	[7,8,34,50,55,62]
***Serratia***	*marcescens*	[7,8,55,58]
***Acinetobacter***	*baumannii*	[7,52,61]
***Klebsiella***	*pneumoniae*	[7,34,36,37,55,58,64]
	*oxytoca*	[7]
***Morganella***	*morganii*	[7,60,61]
***Propionibacterium***	*acnes*	[7,36,60]
***Stenotrophomonas***	*maltophilia*	[7,55,62]
***Bacteroides***	*fragilis*	[7,60,62]
***Aeromonas***	*hydrophila*	
***Alcaligenes***	*faecalis*	[7]
***Bacillus***	*cereus*	
***Citrobacter***	*koseri*	[7,34]
	*freundii*	[7,55]
***Clostridium***	*perfringens*	[7,36]
***Listeria***	*monocytogenes*	
	*inocua*	
***Peptostreptococcus***	*anaerobius*	[7]
***Veillonella***	*parvula*	
